# Exploring the lipids, carotenoids, and vitamins content of *Rhodotorula glutinis* with selenium supplementation under lipid accumulating and growth proliferation conditions

**DOI:** 10.1186/s12866-024-03585-x

**Published:** 2024-11-06

**Authors:** Nora Elfeky, Aya Rizk, Mohamed M. Gharieb

**Affiliations:** Botany and Microbiology Department, Faculty of Science, Menoufia University, Menoufia, Egypt

**Keywords:** *Rhodotorula glutinis*, Selenium, Lipids, Cartenoids, Vitamins, Transmission electron microscope

## Abstract

**Background:**

*Rhodotorula glutinis*, a specific type of yeast, has been recognised as a superior resource for generating selenium-enriched biomass that possesses exceptional nutritional and functional attributes. The purpose of this investigation was to assess the effect of sodium selenite at different concentrations on lipid and carotenoid synthesis, as well as the growth of *R*. *glutinis*.

**Methods:**

The lipid’s fatty acid composition was determined using gas chromatography (GC). The vitamins were detected by high-performance liquid chromatography (HPLC). Transmission electron microscopy was used to detect the structural modification of yeast cells caused by the addition of sodium selenite to the growth medium, as well as the accumulation of elemental selenium in the yeast cells.

**Results:**

The yeast cells demonstrated the ability to endure high concentrations of sodium selenite under lipid accumulation (LAM) and growth-promoting (YPD) conditions. 25.0 mM and 30.0 mM, respectively, were published as the IC50 values for the LAM and YPD conditions. In both growth media, 1 mM sodium selenite boosted lipid synthesis. Lipid accumulation increased 26% in LAM to 11.4 g/l and 18% in YPD to 4.3 g/l. Adding 1 mM and 3 mM sodium selenite to YPD medium increased total and cellular carotenoids by 22.8% (646.7 µg/L and 32.12 µg/g) and 48.7% (783.3 µg/L and 36.43 µg/g), respectively. Palmitic acid was identified as the most abundant fatty acid in all treatments, followed by oleic acid and linoleic acid. The concentrations of water soluble vitamins (WSV) and fat soluble vitamins (FSV) were generally significantly increased after supplementation with 1.0 mM sodium selenite. TEM examination revealed a significant reduction in lipid bodies accumulation in the yeast cells when sodium selenite was added to lipid-promoting environments. This decline is accompanied by an augmentation in the formation of peroxisomes, indicating that selenium has a direct impact on the degradation of fatty acids. In addition, autophagy appears to be the primary mechanism by which selenium ions are detoxified. Additionally, intracellular organelles disintegrate, cytoplasmic vacuolization occurs, and the cell wall and plasma membrane rupture, resulting in the discharge of cytoplasmic contents, when a high concentration of sodium selenite (20.0 mM) is added. Also, the presence of numerous electron-dense granules suggests an intracellular selenium-detoxification pathway.

**Conclusion:**

This study proposes the use of YPD with 1 mM sodium selenite to cultivate selenium-enriched biomass from *R. glutinis*. This approach leads to heightened lipid levels with higher accumulation of oleic, linoleic and linolenic acids, carotenoids, and vitamins. Hence, this biomass has the potential to be a valuable additive for animal, fish, and poultry feed. Furthermore, explain certain potential factors that indicate the impact of selenium in reducing the accumulation of lipid droplets in *R. glutinis* during lipogenesis, as detected through TEM examination.

**Supplementary Information:**

The online version contains supplementary material available at 10.1186/s12866-024-03585-x.

## Introduction

In recent years, microbial technology has attracted considerable attention from researchers owing to its uses across diverse industries, including its function as antimicrobial agents [1, 2], a source of vital chemicals [3], and for the production of lipids and carotenoids [4, 5]. *Rhodotorula glutinis* (*R. glutinis*), an oleaginous pigmented yeast, has garnered significant interest as a possible source of biomolecules. The aforementioned organism is noted for its capacity to produce significant amounts of lipids and carotenoids, which have many applications in industries like as food, cosmetics, and biofuel [4, 5]. Under some circumstances, *R. glutinis* have the ability to accumulate lipids into structures known as lipid bodies (LB), which can constitute as much as 65% of their total dry biomass [5, 6]. Their fatty acids are primarily characterised by the prevalence of oleic, linoleic, and palmitic acid [6]. Oleaginous yeast lipids can be utilized to make biodiesel when enriched with saturated fatty acids such as palmitic acid and stearic acid [7]. It may have applications in the food industry and therapeutic contexts when enriched with polyunsaturated fatty acids such as linoleic acid and linolenic acid [7]. Furthermore, owing to its elevated lipid profile akin to that of plants, it may be employed in the manufacture of animal or fish feed [8].

In addition, *R. glutinis* possess the ability to biosynthesize carotenoids, including β-carotene, torulene, and torularhodin [6]. Carotenoids are frequently employed in the cosmetic, pharmaceutical, and food sectors due to their advantageous properties that promote health [1]. Additionally, they are employed as supplementary substances in animal feeds for livestock, fish, and crustaceans [5].

Selenium is a micronutrient that is essential for the proper functioning of all organisms and maintaining human health. Selenium deficiency affects approximately 0.5–1 billion people around the world and is prone to causing health disorders such as cancer, Keshan disease, Kashin-Beck disease, muscular syndrome, and even death [9, 10]. In order to mitigate the risk of selenium insufficiency, it is advisable to consider the implementation of selenium supplementation in both agricultural lands and the feeding of animals, poultry, and fish [11]. Consequently, it can be administered in an appropriate quantity within the human dietary intake [11]. To address the potential deficiency of selenium (Se) and meet the Se requirements of livestock animals, feed supplementation is commonly employed. This involves the addition of Se in various forms, including inorganic forms such as sodium selenite (SS) and soya protein hydrolysates [12]. Additionally, organic forms such as Se-yeast (SY), which is yeast enriched with selenium methionine (SeMet) and selenium cytosine (SeCys) [12]. Selenium-enriched yeast has been widely regarded as a secure and efficacious means of fortifying nutrients among the diverse array of selenium-enriched goods. Prior research has confirmed that the inclusion of selenium-enriched yeast in one’s diet significantly enhances overall health in comparison to the use of inorganic selenium products [13].

According to recent research, it has been demonstrated that *Rhodotorula glutinis* possesses the capacity to accumulate selenium within its biomass [14]. Consequently, it has the potential to function as a source of selenium as well. Although *R. glutinis* is an important source of pigments, lipids, many essential vitamins, and valuable compounds, few scientists have looked into how selenium affects the biosynthesis of pigments, lipids, and vitamins within yeast cells. In addition, there is a clear gap in the research concerning the optimal conditions for sequestering selenium within oleaginous pigmented yeasts. It is well established that a high carbon to nitrogen ratio, coupled with a limited nitrogen source in the culture media, promotes lipid formation in oleaginous yeasts [15–17]. However, these conditions also hinder cell proliferation and biomass generation [6] So, the question becomes, are conditions that favour the accumulation of lipids or conditions that favour the proliferation of oleaginous yeast best promote selenium accumulation within the yeast?

The objective of this study is to investigate the impact of varying selenium concentrations on the synthesis of lipids and carotenoids in both lipid-accumulating (LAM) and growth-proliferating (YPD) conditions. Furthermore, examine the influence of selenium on the composition of fatty acids and vitamins in *R. glutinis* is being conducted. Moreover, investigate the alterations in the cellular structure of yeast cells following exposure to selenium in conditions that promote lipid accumulation. This study lays the basis for finding the ideal circumstances under which *R. glutinis* might be cultivated to be a viable source of organic selenium supplementation.

## Materials and methods

### Yeast strain and cultivation media

The oleaginous red yeast *Rhodotorula glutinis* (*R. glutinis*) (AS 2.703) was used in this study. It was grown for three days at 30 ^o^C on yeast peptone dextrose (YPD) agar slants (glucose 20 g/L, peptone 10 g/L, yeast extract 10 g/L, and agar 15 g/L, pH 5) and then stored at 4 ^o^C until used. We used YPD as a seed culture and as a growth proliferating medium (YPD). Lipid accumulating medium (LAM) contains (per litre) glucose (40 g), (NH_4_)_2_SO_4_ (0.5 g), yeast extract (0.75 g), KH_2_PO_4_ (1.5 g), and MgSO_4_.7H_2_O (1 g). LAM and YPD were used to test the toxicity of different doses of sodium selenite on *R. glutinis* growth, lipids, carotenoids, and vitamins production.

### Testing the growth inhibitory rate of sodium selenite

Newly cultivated yeast cells were inoculated into YPD broth (100 ml in 250 ml conical flasks), which was then incubated to the mid-log phase (OD 600 ≈ 1.0). At that time, cells were collected by centrifugation at 3000 rpm for 5 min, washed twice with sterile water, resuspended in LAM and YPD containing different concentrations of sodium selenite (Na_2_SeO_3_ (0.0, 1 mM, 3.0 mM, 5.0 mM, 7.0 mM, 10.0 mM, 15.0 mM, 20.0 mM, 25.0 mM, 30.0 mM, and 35.0 mM). After three days of incubation at 30 °C in the dark, suitable dilutions were plated on yeast extract peptone dextrose agar plates and the number of colony-forming units (CFU) was determined. The inhibitory ratio was calculated with the following formula:

$$\:Inhibitory\:ratio\:\text{\%}=\frac{CFU\:OF\:CONTROL-CFU\:of\:treated\:group}{CFU\:of\:the\:control}\times\:100$$

The IC50 value shows the inhibitor concentration at which 50% inhibition occurs.

### Estimation of dry cell weight (DCW), total lipids (TL), and total carotenoids (TC)

To find out how different sodium selenite concentrations affect dry cell weight, lipids, and carotenoids production, LAM and YPD with different sodium selenite concentrations (as mentioned above) were inoculated with yeast cells and incubated for 5 days at 30 ^o^C and 120 rpm. DCW was detected as stated by Elfeky et al. [5]. The well-washed yeast cells from 5 ml were freeze-dried for 24 h until the constant weight was recorded and the final DCW was determined. The total lipids in yeast cells were measured using the sulfo-phospho vanillin method [18], which Elfeky et al. [5] summarised briefly.

To extract carotenoids from yeast cells, 10 mL cultures were centrifuged for 10 min at 6000 rpm, followed by three washes with sterile distilled water. The yeast cells were then hydrolyzed by boiling for 5 min in 1 mL of 1 M HCl, followed by centrifugation at 6000 RPM for 10 min, and then washing to neutral pH with pure H_2_O. The hydrolyzed cells were progressively mixed with 1 mL acetone, followed by 0.5 mL ethyl acetate, as solvent was added gradually. This technique was continued until the carotenoid extraction was complete. The solvent mixture was vacuum-dried prior to dissolution in 1 mL acetone. For further investigation, the dissolved carotenoid was filtered through a 0.45 μm. All prior procedures were performed in low-light circumstances to prevent pigment deterioration. The spectrophotometer reading at 485 nm was used to get the total carotenoids content [5]. 

### The effect of sodium selenite on the fatty acid profile of *Rhodotorula glutinis* grown in LAM

Transesterification of the extracted lipid samples from the yeast cells cultivated in LAM supplemented with 1mM sodium selenite was performed as described by Van Wychen et al. [37] and briefly summarised by Elfeky et al. [6] to determine the fatty acid composition. An Agilent 7890B GC equipped with an autosampler and flame ionisation detector (FID) was used to analyse the fatty acid methyl ester. A Zebron ZB-FAME column (60 m x 0.25 mm internal diameter x 0.25 m film thickness) was used for separation. Analyses were performed with hydrogen as the carrier gas at a flow rate of 1.8 ml/min in a split-1:50 mode, an injection volume of 1 µl, and the following temperature programme: 100 °C for 3 min; rising at 2.5 °C/min to 240 °C and holding for 10 min. The injector and detector (FID) were kept at 250 and 285 degrees Celsius, respectively. Fatty acids were identified by comparing their retention times to those of standard ones and were quantified as a percentage of the total FAMES content.

### Water soluble vitamins (WSV) and fat soluble vitamins (FSV) extraction and detection

To detect the effect of 1.0 mM of sodium selenite concentrations on the accumulation of WSV and FSV in *R. glutinis* under lipogenesis and non-lipogenesis conditions. The yeast cultures were inoculated with yeast cells and incubated for 5 days at 30 ^o^C and 120 rpm. The vitamin extract from the yeast biomass were prepared according to method described by Abe-Matsumoto et al. [19], and summarized briefly by Assis et al. [20].

HPLC analysis was carried out using an Agilent 1260 series. The separation was carried out using ZORBAX SB-C8 (4.6 mm x 150 mm i.d., 5 μm). The mobile phase consisted of water with 0.01% TFA (pH 2.9) (A) and Methanol (B) at a flow rate of 1.5 ml/min. The mobile phase was programmed in the case of water-soluble vitamins consecutively in a linear gradient as mentioned in Table 1 and the injection volume was 5 uL. The multi-wavelength detector was monitored at 280 nm.

**Table 1:** Mobile phases of the programe used for the separation of water-soluble vitamins in HPLC

Time (min)	A (%)	B (%)	Flow (mL/min)
0	90	10	1.5
1	70	30	1.5
4	50	50	1.5
8	90	10	1.5
10	90	10	1.5

For fat-soluble vitamin, the column used was Agilent C18 (4.6 mm x 100 mm i.d., 3.5 μm). The mobile phase was methanol: acetonitrile 65:35 and the flow rate was 1 mL/min. The injection volume was 20 µl for each of the sample solutions. The DAD was adjusted at 295, and 254 nm. The Fluoresence detercot was adjusted at 290/330 nm (Excitation/Emission). The column temperature was maintained at 40 °C.

### Transmission electron microscope examination

The yeast cells were cultivated on YPD and LAM media. LAM supplemented with varying concentrations of sodium selenite (0 mM, 10 mM, and 20 mM), for a period of three days. Following that, the cells were collected, rinsed with double distilled water, and subsequently exposed to an overnight fixation process in a chilled solution containing 2.5% glutaraldehyde, prepared in a 0.1 M potassium phosphate buffer with a pH value of 7.4. Subsequently, the specimens underwent a postfixation process lasting 3 h, during which they were immersed in a 1% solution of osmium tetroxide (OSO_4_) in the same buffer. The materials were subjected to an overnight staining process and afterwards dehydrated following the protocol outlined by Wright [21]. The stained sections were examined by transmission electron microscope (TEM Joel- 1400) at the candidate magnification. At least 10 sections of each of the prepared samples were examined in The Electron Microscope Unit, Faculty of Agriculture, Research Park, Cairo University, (FARP). Electron micrographs were captured using CCD camera model AMT, Optronics camera with 1632 × 1632 pixel format as side-mount configuration.

### Statistic analysis

Results are expressed as means of different experiments ± standard deviation (SD). Results were statistically analyzed using One-way ANOVA was performed using GraphPad Prism version 6.04 for Windows, GraphPad Software, www.graphpad.com. (*) means 0.01 < *P* < 0.05, (**) means 0.001 < *P* ≤ 0.01, (***) means *P* ≤ 0.001.

## Result and discussion

### Toxicity of different sodium selenite concentrations on *Rhodotorula glutinis* under lipogenesis and non-lipogenesis conditions

**Fig. 1:**
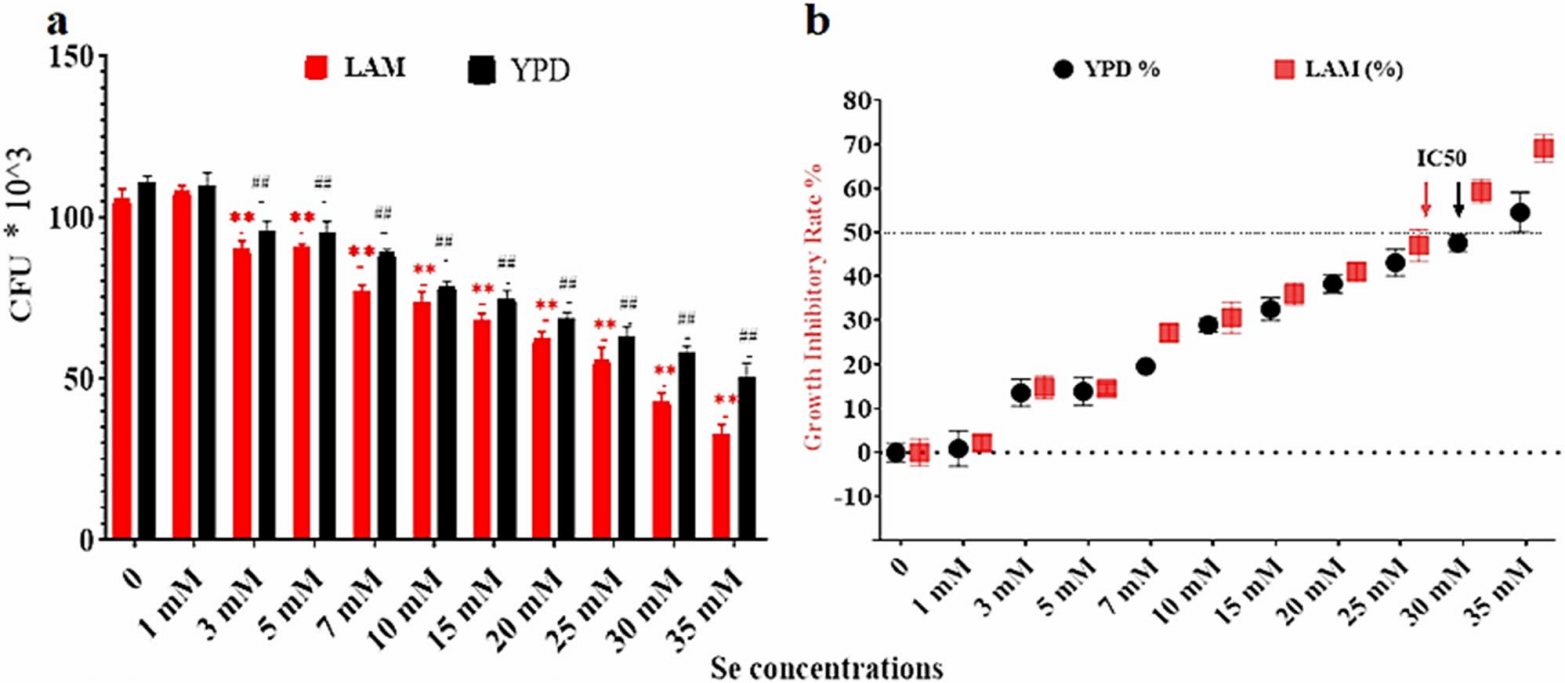
Influence of Sodium Selenite Concentrations on *Rhodotorula glutinis* Growth in Lipogenic (LAM) and Non-Lipogenic (YPD) Conditions after three days. (**a**) shows the colony forming units (CFU) per milliliter under the tested conditions, (**b**) the growth inhibitory rate of sodium selenite on *R. glutinis*. The IC50 value, represented by an arrow, designates the sodium selenite concentration at which 50% growth inhibition is observed. Data are averages derived from three biological replicates with error bars denoting standard deviation. (**) & (##) mean 0.001 < *P* ≤ 0.01, (***) & (###) mean *P* ≤ 0.001

Our study investigated the physiological behavior of *R. glutinis* under various sodium selenite concentrations. We tested sodium selenite concentrations ranging from 1 mM to 35 mM in both YPD and LAM media to determine the potential toxicity of sodium selenite on *R. glutinis* growth. Our findings show a dose-dependent inhibitory effect of sodium selenite on the growth of *R. glutinis* in both YPD and LAM media over a three-day period (Fig. 1a). A significant decrease in the number of viable cells was observed at a sodium selenite concentration of 3 mM, indicating the onset of toxicity at this concentration. A drastic decrease in the number of viable cells was noted at 35 mM, highlighting the high toxicity of sodium selenite at this concentration.

The IC50 value, which is the concentration of an inhibitor where the response is reduced by half, was found to be lower for cultures grown in LAM-Se (25 mM) than for those in YPD-Se (30 mM) (Fig. 1b). The different IC50 values in YPD and LAM suggest that the growth media can influence the yeast’s sensitivity to sodium selenite.

The result suggests that Selenite ions (Se) manifest a higher toxicity to yeast during lipid-accumulating conditions compared to normal cell proliferation conditions (YPD). The sensitivity of *Rhodotorula* species to selenium has not been extensively studied according to the existing literature. On the other hand, research involving other yeast species has demonstrated that an excess of selenium can obstruct the enzymatic oxidation of yeast macromolecules [22]. Se has also been reported to inhibit *Candida utilis* ATCC 9950 and *Saccharomyces cerevisiae* ATCC MYA-2200 [23], and *Yarrowia lipolytica* [24]. Numerous researchers have used *S. cerevisiae* as a model microorganism to examine the toxicity and mutagenicity of Se and/or other metalloid elements [25]. According to the published literature, Se damages double-stranded DNA in *S. cerevisiae* [26, 27] and reduce of glutathione (GSH) [26]. Furthermore, elevated concentrations of selenium, especially in its selenide form, are notably toxic to cells, leading to the production of reactive oxygen species (ROS) [28]. These ROS can induce oxidative damage to proteins, lipids, and DNA, potentially causing cell death. We propose that the metabolic status of cells during lipogenesis could account for the toxicity of selenium on the yeast compared with the YPD. In this phase, cells experience stress due to the accumulation of free fatty acids and the formation of lipid droplets, both of which amplify oxidative stress. This might explain the higher toxicity of selenium seen during lipogenesis when compared with normal growth conditions.

### Effect of selenite on the DCW, lipids content and carotenoids content of *R. Glutinis*

**Fig. 2:**
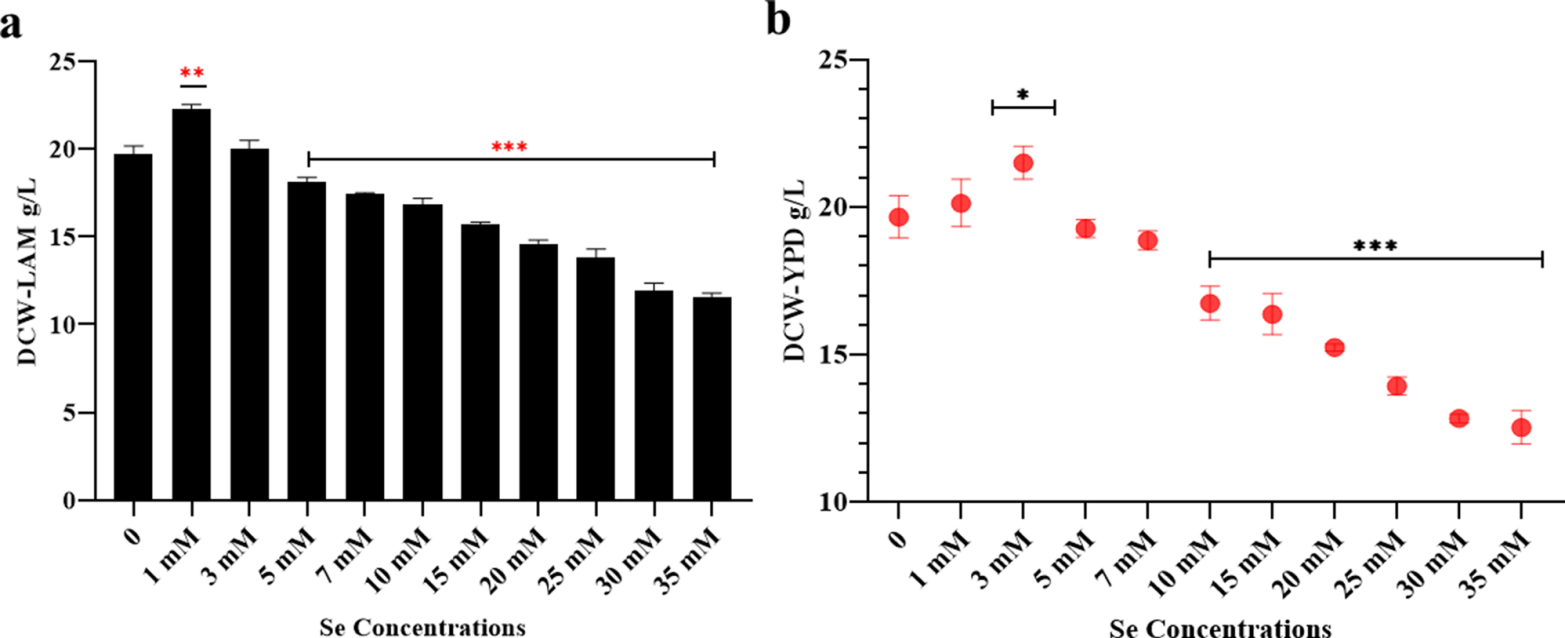
Effect of different concentrations of sodium selenite on dry cell weight of *R. glutinis* under different media composition incubating at 30 ^o^c for five days: (**a**) lipid accumulating condition (LAM), and (**b**) growth proliferating condition (YPD). Data are averages derived from three biological replicates with error bars denoting standard deviation. * means 0.05 > *p* > 0.01, ** means 0.01 ≥ *p* > 0.001, and *** means *p* ≤ 0.001

To examine the effect of sodium selenite on the dry cell weight (DCW), lipids, and carotenoids production of *Rhodotorula glutinis*, various sodium selenite concentrations were added to limited nutrient media (LAM) and nutrient rich media (YPD). As shown in Fig. 2, regardless of the culture media employed, dry cell weight was substantially increased at 1 mM and 3 mM sodium selenite (Fig. 2a, b), whereas further increasing sodium selenite in the culture media resulted in a significant decrease in biomass production. Recently, a study conducted by Zhang et al. [29] demonstrated that the presence of Se hinders the cellular growth of *Candida utilis* CCTCC M 209,298. The researchers proposed that Se reduces carbon fluxes directed towards cell mass formation while increasing fluxes towards amino acid production for creating glutathione and associated amino acids. The majority of studies on Se-enriched yeasts are focused on its integration into proteins, replacing sulfur, and its influence on their structure and catalytic functions [30]. El-Bayoumy et al. [31] explored the impact of Se on the comprehensive protein expression of Se-enriched *S. cerevisiae*. Their findings revealed that Se led to the upregulation of several proteins, including pyruvate kinase, elongation factor 2, and heat shock protein 70.Conversely, Triosephosphate isomerase (TSI) was downregulated. TSI is a crucial enzyme in glycolysis and is therefore vital for energy production. According to the authors, Se impacts a range of metabolic processes, including glycolysis, ATP binding, metal binding, nucleoside and nucleotide binding, and protein structure.

**Fig. 3:**
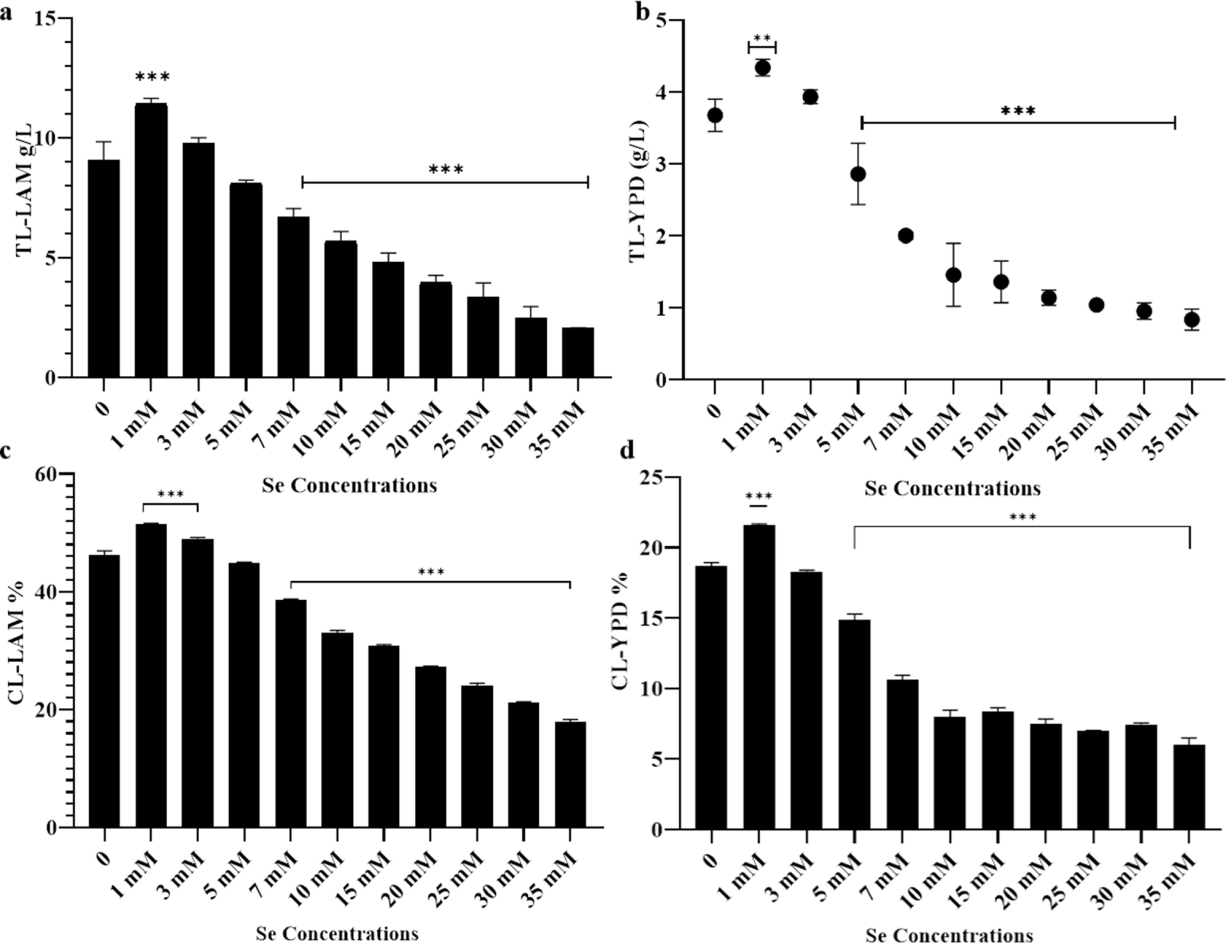
Effect of different concentration of sodium selenite on lipids accumulation in *R. glutinis* in different culture media for five days at 30 ^o^c; (**a**) total lipids in LAM,. (**b**) total lipids content in YPD, (**c**) cellular lipids in LAM, (**d**) cellular lipids in YPD. Data are averages derived from three biological replicates with error bars denoting standard deviation. (**) means 0.001 < *P* ≤ 0.01, (***) means *P* ≤ 0.001

Introducing 1 mM sodium selenite significantly boosted lipid accumulation in both types of culture media. Specifically, the lipid accumulation increased approximately 26%, reaching 11.4 g/l in LAM and 18% in YPD (4.3 g/l) (Fig. 3a, b). In the LAM, the cellular lipid percentage was found to be 51.4% at a concentration of 1 mM, in comparison to 46.2% in the control group, as evidenced in Fig. 3c. Further supplementation with sodium selenite resulted in a marked decrease in lipid accumulation under both lipogenesis and non-lipogenesis conditions (Fig. 3).

A comparable outcome was documented in a recent investigation that examined the impact of a concentration of 10 mg/L of sodium selenite on the proliferation and lipid accumulation of *Yarrowia lipolytica* [29]. The researchers discovered that the introduction of selenium supplementation resulted in a reduction in both lipid and biomass production. The potential suppression of lipid accumulation could be associated with the stimulation of reactive oxygen species (ROS) generation, which is a consequence of the harmful effects of selenium (Se) in the culture medium. The accumulation of reactive oxygen species (ROS) has the potential to initiate lipid peroxidation, hence leading to the disruption of the cytoplasmic membrane’s structural integrity [32].

**Fig. 4:**
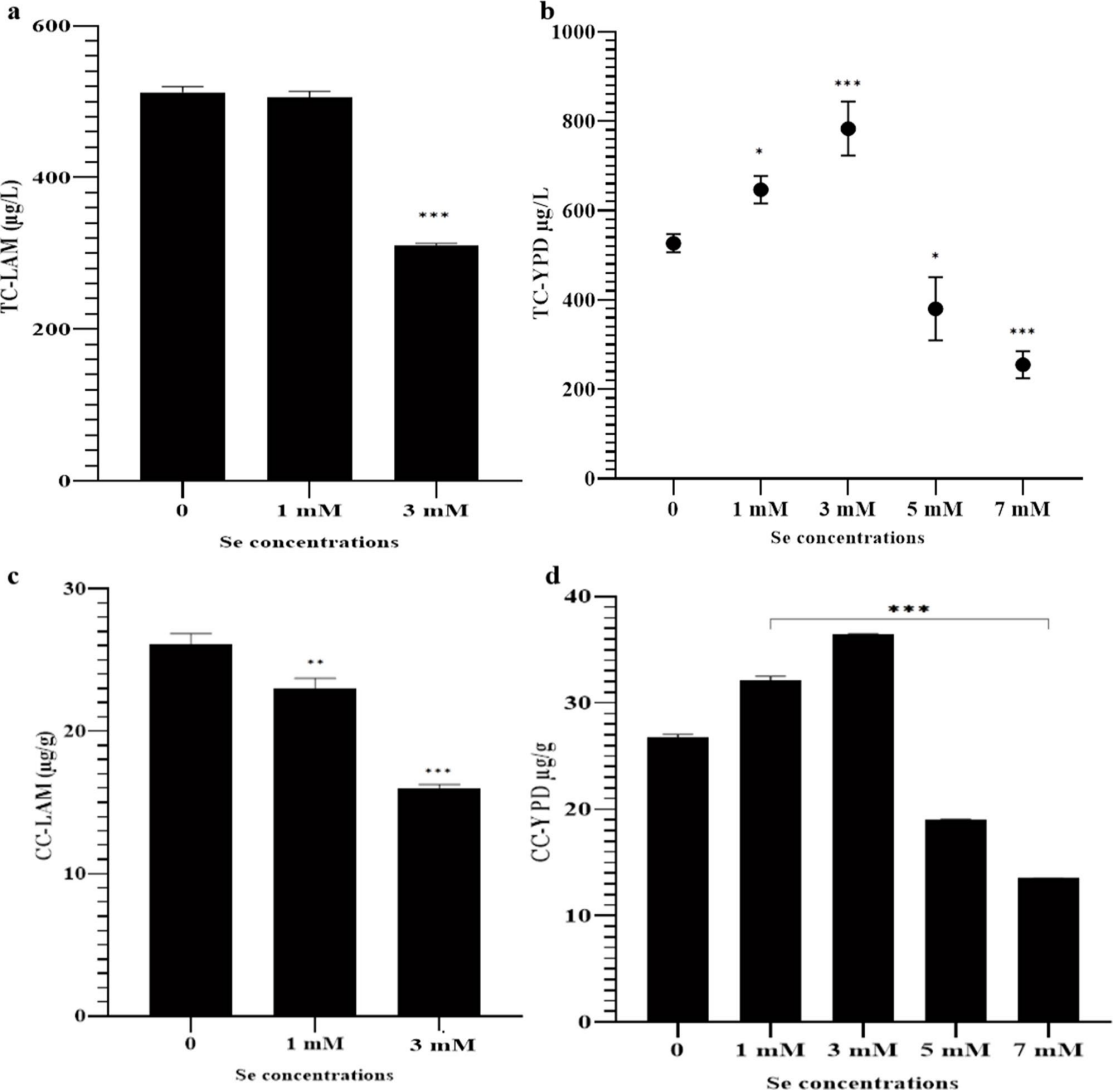
Effect of different concentrations of sodium selenite on carotenoids accumulation in *R. glutinis* cultivated in different culture media for five days at 30^o^c; (**a**) total carotenoids under lipogenesis (LAM), (**b**) total carotenoids under non-lipogenesis (YPD) conditions, (**c**, **d**) cellular carotenoids in LAM and YPD, respectively. Data are averages derived from three biological replicates with error bars denoting standard deviation. * means 0.05 > *p* > 0.01, ** means 0.01 ≥ *p* > 0.001, and *** means *p* ≤ 0.001

In contrast, the concentrations of both total carotenoids and cellular carotenoids experienced a significant increase of around 22.8% (646.7 µg/L, and 32.12 µg/g, respectively) and 48.7% (783.3 µg/L, and 36.43 µg/g, respectively ) when subjected to treatment with 1 mM and 3 mM of sodium selenite in YPD medium, respectively. Nevertheless, when the dose reached 10 mM, there was a significant decrease in carotenoid levels, resulting in complete suppression (Fig. 4b, d). The addition of selenium has been observed to negatively affect the synthesis of carotenoids during the process of lipogenesis. The findings indicate that carotenoid production is completely suppressed when the concentration reaches 5 mM, as depicted in Fig. 4 (a, c).

In a comparable investigation conducted by Breierova et al. [33], the impact of selenium supplementation on carotenoid synthesis by *Rhodotorula glutinis* and *Sporobolomyces roseus* cultivated on malt extract media was examined. The researchers investigated the impact of several amounts of sodium selenite (20 mg/L, 40 mg/L, 60 mg/L, 80 mg/L, and 100 mg/L) on the growth of yeast strains and the generation of carotenoids. The findings of the study indicated that the inclusion of selenium in the culture media resulted in a notable reduction in cellular carotenoid levels, particularly β-carotene.

Numerous studies have documented the augmentation of volumetric production and cellular accumulation of microbial carotenoids with the introduction of metal ions as copper, zinc, and aluminium at the growth media [6, 34, 35]. The carotenoid profile in red yeasts has been observed to be selectively influenced by trace elements. The phenomenon can be elucidated by postulating potential mechanisms of activation or inhibition by certain metal ions on specific enzymes involved in carotenoid synthesis [34]. A recent study using multi-omics metabolic analysis to investigate the mechanisms behind the development of stress resistance in *R. glutinis* induced by irradiation. The study’s findings confirmed a significant enhancement in the expression of the carotenoid biosynthesis pathway. This suggests that the increased carotenoid concentration functions as a cellular defense mechanism against oxidative stress caused by irradiation [36]. In relation to selenium, it is hypothesised that an appropriate concentration of selenium could potentially augment the production of carotenoids, taking into account the composition of the culture media and the conditions of cultivation. However, it should be noted that elevated levels of selenium, which reach toxic thresholds, may have the potential to impede the activity of diverse enzymes and precursors involved in the synthesis of both primary and secondary metabolites. Consequently, this may have a detrimental impact on the final biomass, lipid content, and yield of carotenoids following the cultivation process.

### The effect of sodium selenite on the fatty acid profile of *Rhodotorula glutinis* grown in LAM and YPD

According to the result obtained from the effect of sodium selenite on both volumetric and cellular content of lipid production by *R. glutinis*. 1 mM was found to be the best concentration to enhance the lipids production. So, the fatty acid profile of the lipids extracted from *R. glutinis* growing on lipid accumulating media (LAM) and growth proliferating media (YPD) treated with 0, and 1 mM of sodium selenite was detected by GC (Table 2). The result showed that the dominant fatty acids in all treatments was palmitic acid followed by oleic acid and linolineic acid, according to the statistical analysis, all fatty acids showed a significant difference (*p* < 0.001) between groups. What ever the used culture media was, 1 mM of sodium selenite lead to increase the ratio of unsaturated fatty acids on behalf of the saturated profile with an obvious increase in growth proliferating condition over lipid accumulation ones (Table 2). Our result was in agreement with a recent published paper, the authors found that the supplemintation of selenium in the culture media of the red pigmented yeast was signiffecantly enhanced the accumulation of unsaturated fatty acids especially oleic and linoleic acid. The authors suggested that the biosynthesis of C18 unsaturated fatty acids in *Rhodotorula* and *Sporobolomyces* species may be associated with phosphatidylcholine moieties, selenium might be involved to the induction of membrane bound fatty acid ∆12 and ∆15 desaturases in red yeasts [37].

Similar results were observed in animal model where both concentration of polyunsaturated fatty acids (mainly arachidonic acid) and antioxidant status increased in lambs fed by Se combined with sunflower oil [38]. Se deficiency also significantly reduced levels of n-3 fatty acids and other C20-C22 polyunsaturates in rat liverphospholipids [39]. Because selenized yeasts, mainly *S. cerevisiae*, have been used for several applications [40, 41], our findings might be very useful for preparation of selenized red yeasts containing carotenoid pigments with enhanced accumulation of linoleic and linolenic acids.

**Table 2:** Ratios of fatty acids fractions of *R. Glutinis* grown on YPD and LAM with and without sodium selenite

	RT	YPD	YPD-Se	LAM	LAM-Se
Caprylic acid (C8:0)	**8.055**	**1.60 ± 2.2** ^**a**^	**0.88 ± 1.2** ^**e**^	**4.42 ± 0.6** ^**i**^	**1.85 ± 1.7** ^**n**^
Lauric acid (C12:0)	**17.562**	**3.20 ± 0.9** ^**b**^	**1.52 ± 2.4** ^**f**^	**3.72 ± 1.8** ^**j**^	**5.11 ± 1.2** ^**o**^
Myristic acid (C14:0)	**23.531**	**4.30 ± 2.3** ^**c**^	**2.60 ± 0.8** ^**g**^	**1.41 ± 1.8** ^**k**^	**1.84 ± 1.8** ^**p**^
Palmitic acid (C16:0)	**29.43**	**36.4 ± 4.8** ^**abcd**^	**33.0 ± 3.6** ^**efgh**^	**38.5 ± 5.3** ^**ijklm**^	**37.4 ± 4.9** ^**nopq**^
Stearic acid (C18:0)	**35.002**	**13.2 ± 1.2** ^**abd**^	**11.6 ± 2.1** ^**efg**^	**19.7 ± 3.1** ^**ijkm**^	**18.4 ± 1.8** ^**nopq**^
Oleic acid (C18:1)	**35.836**	**20.4 ± 1.9** ^**abc**^	**25.3 ± 1.9** ^**efgh**^	**18.8 ± 2.7** ^**ijkm**^	**21.1 ± 1.2** ^**nopq**^
Linoleic acid (C18:2)	**37.534**	**15.3 ± 2.4** ^**abc**^	**17.7 ± 1.2** ^**efgh**^	**10.5 ± 1.7** ^**l**^	**11.7 ± 2.3** ^**np**^
Linolenic acid (C18:3)	**39.639**	**5.60 ± 2.4** ^**d**^	**7.40 ± 1.8** ^**h**^	**2.90 ± 0.8** ^**lm**^	**3.17 ± 0.7** ^**q**^
p-value	**< 0.001**				

The result are the mean of three replica ± standard deviation. The data with the same letter are significantly different (*p* < 0.001)

### Estimating the impact of varying sodium selenite concentrations on yeast vitamins content under lipogenesis and non-lipogenesis conditions

**Fig. 5:**
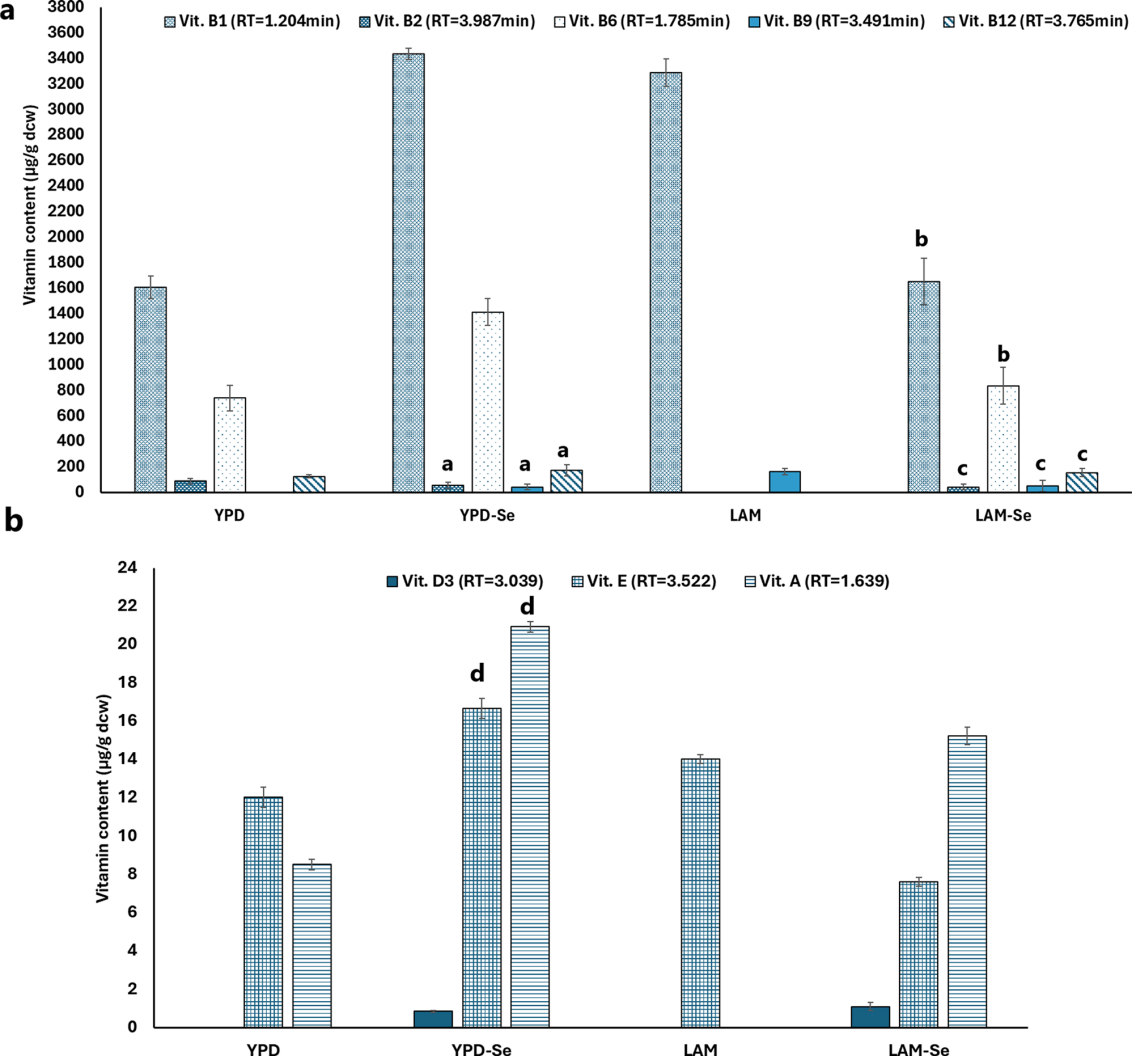
Concentrations of water soluble vitamins (WSV) fractions (**a**) and fat soluble vitamins (FSV) (**b**) of *R. glutinis* grown on YPD and LAM with and without sodium selenite. The series with the same letter are not significant difference (*p* > 0.05). The anova analysis showed a significant difference between groups (*p* < 0.001)

To estimate the possibility of producing selenized red yeasts, we study the effect of 1 mM of sodium selenite on the vitamins content in *R. glutinis*. Figure 5 represents the variation in the water soluble vitamins (WSV) fractions and fat soluble vitamins (FSV) of *R. glutinis* grown on YPD and LAM with and without sodium selenite. In the case of WSV, both media experience a general increase following the addition of Se, with the exception of Vit. B2 with YPD, which experiences a roughly 36% decrease, and Vit. B1 with LAM, which experiences a 49.7% decrease. While the other WSV experience a rise of over 90% following selenium supplementation (Fig. 5a). Additionally, the FSV (Vit.D3, Vit. E, Vit. A) exhibited a significant increase in both media (Fig. 5b). Zhang et al. [42] reported that selenium supplementation improved the regulation of amino acids in mice. Other study has also shown that systematically omitting single amino acids can greatly affect the production of B12 in *Lactobacillus reuteri* [43]. For example, omitting isoleucine resulted in nearly undetectable levels of B12, while omitting cysteine led to levels 20 times higher than previously reported. As a result, we hypothesised that selenium addition in the yeast culture could have a direct or indirect impact on the amino acids pathways, hence affecting vitamins production.

### Ultrastructural variation after growth of *Rhodotorula glutinis* on LAM with different concentrations of sodium selenite in *R. glutinis* cells

**Fig. 6:**
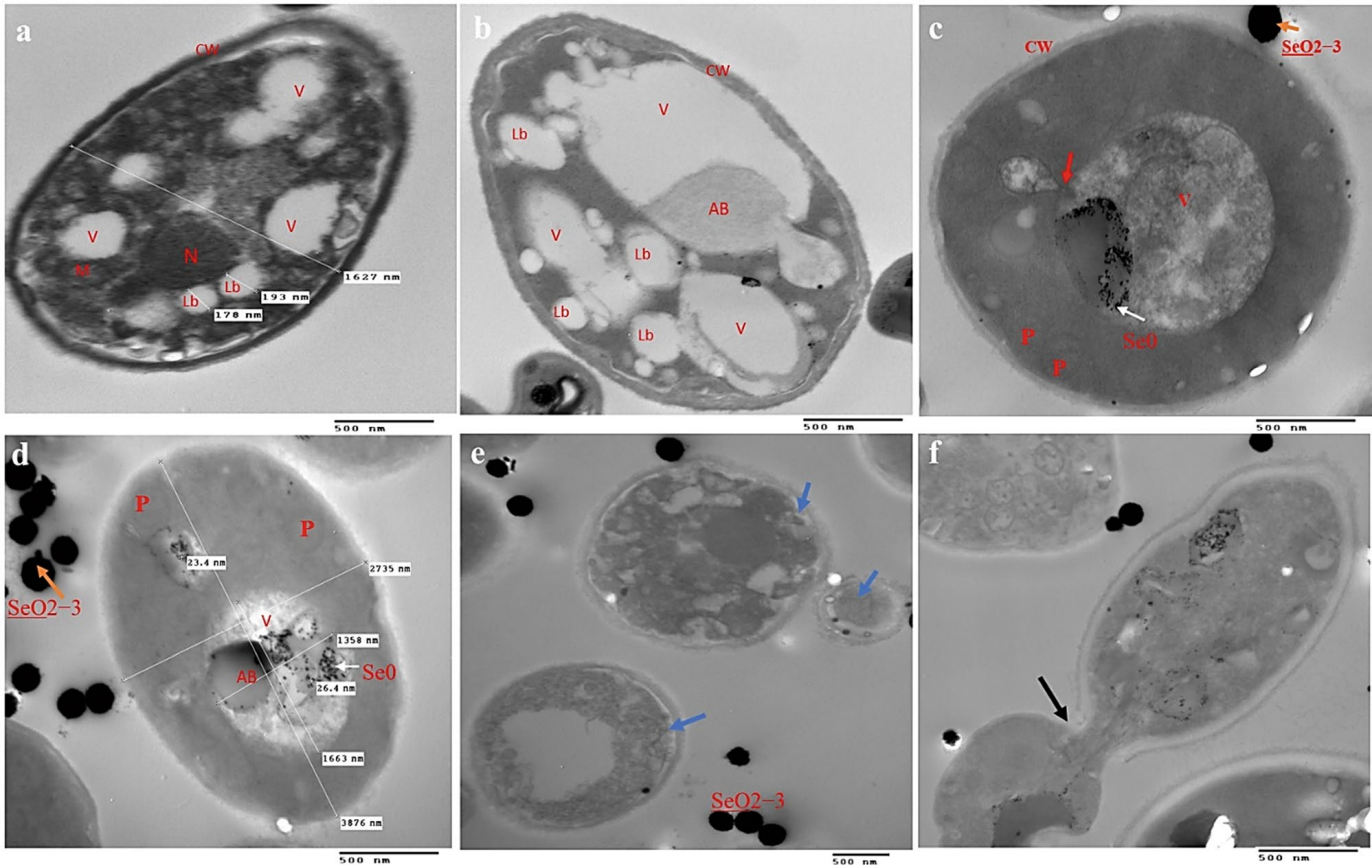
Ultrastructural variation after growth of *Rhodotorula glutinis* on different culture media for three days: (**a**) YPD, (**b**) LAM, (**c**) LAM-containing 10 mM Selenite, and (**d**-**f**) LAM-containing 20 mM Selenite. Scale bar is equal to 500 nm. V, Vacoule; N, nucleus; m, mitochondria; CW, cell wall; AB, autophagic body; lb, lipid body; P,peroxisomes

The study involved examining the structural changes in *R. glutinis* when subjected to lipid accumulation conditions and treated with different doses of sodium selenite. This examination was carried out using transmission electron microscopy, as depicted in Fig. 6. During the growth on the nutrient rich medium (YPD), *R. glutinis* was able to form yeast cells, which were round or slightly ellipsoidal, while lipid droblets (LDs) were clearly visible and they had different shapes and sizes (Fig. 6a). Growing the yeast cells in LAM conditions lead to increase the thickness of the cell wall compared to the YPD media (Fig. 6b). One particularly intriguing phenomenon is the emergence of autophagic bodies (ABs) in the form of vesicles within the vacuole (Fig. 6b). Autophagy is a crucial metabolic process that cells undergo in response to starvation in order to sustain viability during food deprivation or eliminate unwanted components within the cells [44]. Hence, organelles have the potential to undergo nonselective autophagy, as well as being subject to particular degradation mechanisms [45]. Selective organellar autophagy can be triggered by organelle injury or failure, as well as by cellular adaptability to fluctuating nutritional circumstances [45].

Following the introduction of sodium selenite at concentrations of 10 and 20 mM into the LAM, an investigation was conducted to examine the impact of these additions on both lipid accumulation and cell structure. When exposed to a concentration of 10 mM sodium selenite (Fig. 6c, d), the cell wall exhibited a decrease in thickness when compared to the cells grown in YPD and LAM media. Lipid bodies were not commonly found, which could be connected to the high concentration of peroxisomes (P) found in yeast cells (Fig. 6c). This particular organelle is essential to the process by which yeast cells break down fatty acids. Enzymes found in peroxisomes are in charge of breaking down fatty acids and initiating the detoxification process within cells [46]. Also, the vacuoles are abundant in the excretion of autophagic bodies. The autophagosome was seen to bind with the lamina of the central vacuoles, as indicated by the red arrow. This suggests that the cells are attempting to survive in an environment with a high quantity of sodium selenite. Similar observations were made when the quantity of sodium selenite was increased to 20 mM (Fig. 6d-f). In addition to the previously mentioned alterations, the primary stage of ghost cells (GC, blue arrows) formation was observed (Fig. 6e). During the early phase of autolysis, the yeast cells exhibited distinct characteristics such as the presence of periplasmic space, pyknosis processes, and cytoplasmic vacuolization [47]. Moreover, cell membrane breaking and cytoplasm leakage from the cells was observed in yeast cell (Fig. 6g), which consequently explains to the cytotoxicity of selenium on the yeast cells.

Similarly, in a recent study, the scientists found changes in the physical structure of *Yarrowia lipolytica* when selenium was introduced into the growth media. Selenium altered the morphology of the yeast cells, affecting both the dimensions and arrangement of lipid droplets within them [24].

Following the cultivation of *R. glutinis* in media enhanced with selenium, the colour of the yeast culture transitioned from orange to a deep red colour (Supplementary material 1). The yeast’s metabolic activity is accountable for the alteration in colour of the cells, as it was the reduction of selenite ions to elemental selenium that brought about this shift, Kieliszek et al. [32] described a comparable incident. Our study demonstrated that elemental selenium accumulated in the cytoplasm and vacuoles of yeast as small dense granules measuring 14–30 nm in size (Fig. 6c-f). We postulated that this could be one of the yeast mechanisms implicated in selenium detoxification pathway [48].

In summary, the yeast cells’ structural variation caused by exposure to sodium selenite under lipid accumulation conditions leads to a significant decrease in lipid droplets. This decrease is accompanied by an increase in peroxisomes production, suggesting that selenium directly affects the production and accumulation of fatty acids. In addition, autophagy appears to be the primary method for detoxifying selenium ions, as well as converting them into elemental selenium and storing them in the vacuoles and cytoplasm. Furthermore, the high concentration (20 mM) of sodium selenite also leads to the breakdown of intracellular organelles, cytoplasmic vacuolization, and the shattering of the cell wall and plasma membrane, resulting in the leakage of cytoplasm.

## Conclusion

*R. glutinis*, an oily, pigmented yeast, is ideal for animal and fish feeding. Our study examines the physiological effects of sodium selenite on yeast cultures in two environments: one that promotes lipid accumulation and another that promotes growth. Our strain was resistant to selenium supplementation, with IC50 values of 25 mM and 30 mM for LAM and YPD conditions, respectively. In both growth media, 1 mM sodium selenite dramatically increased lipid production. More specifically, lipid buildup increased by 26% in LAM to 11.4 g/l and 18% in YPD to 4.3 g/l. Cellular lipid in biomass collected from LAM medium was 51.4% at 1 mM, compared to 46.2% in the control condition. However the higher sodium selenite supplementation significantly reduced lipid deposition in lipogenesis and non-lipogenesis. Adding 1 mM and 3 mM sodium selenite to YPD medium enhanced total and cellular carotenoids by 22.8% (646.7 µg/L and 32.12 µg/g, respectively) and 48.7% (783.3 µg/L and 36.43 µg/g, respectively), and carotenoids were completely suppressed at 10 mM. While carotenoids production during lipogenesis are negatively affected by sodium selenite addition to the culture media. Carotenoids are totally inhibited when sodium selenite surpasses 3 mM in LAM conditions. *R. glutinis*’ fatty acid composition after adding 1mM sodium selenite to LAM and YPD media showed palmitic acid topping all treatments, followed by oleic and linoleic acids. The ratio of unsaturated to saturated fatty acids increased, especially under growth conditions. With 1 mM sodium selenite, YPD and LAM had considerably higher WSV and FSV levels. We thought selenium supplementation improved the harvested biomass’s nutritional content by increasing lipids (especially oleic, linoleic and linolenic acids), carotenoids, and vitamins. A transmission electron microscope showed that yeast cells collect elemental selenium in cytosol and vacoules. The transmission electron microscope detected structural alterations in yeast cells after high sodium selenite exposure. The most typical effects of selenium poisoning in yeast cells were decreased lipid body formation, increased peroxisomes, autophagy, and cell lysis.

In summary, this research suggests employing growth proliferating media supplemented with 1 mM sodium selenite to produce selenium-enriched biomass from *R. glutinis*. This method results in improved levels of lipids with enhanced accumulation of oleic, linoleic and linolenic acids, carotenoids, and vitamins compared to lipids accumulation conditions. Additionally, elucidate some potential factors that demonstrate the influence of selenium in diminishing lipids droplets accumulation in *R. glutinis* under lipogenesis settings, as observed through TEM analysis.

## Electronic supplementary material

Below is the link to the electronic supplementary material.

Supplementary Material 1

## Data Availability

The datasets used or analyzed during the preparation of the manuscript are available from the corresponding author at reasonable request.

## Abbreviations

GC: Gas Chromatograph
HPLC: High-performance liquid chromatography
LAM: Lipid accumulation Media
YPD: Growth-promoting conditions
WSV: Water soluble vitamins
FSV: Fat soluble vitamins
Se: Selenium
SS: Sodium selenite
