# Supplementary material for: Exploring the lipids, carotenoids, and vitamins content of Rhodotorula glutinis with selenium supplementation under lipid accumulating and growth proliferation conditions
Source: BMC Microbiol. 2024 Nov 6;24:451. doi: 10.1186/s12866-024-03585-x (PMC11539581; doi:10.1186/s12866-024-03585-x)
Supplement: Supplementary file 1 — Supplementary Material 1 [file 12866_2024_3585_MOESM1_ESM.docx]

**
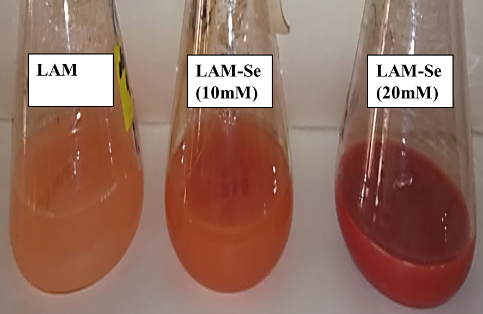
**

**Figure 1** Transition of the yeast culture (aged 3 days) from orange to dark red colour due to sodium selenite reduction to elemental selenium.
